# On the Management of Twin Cases

**Published:** 1811-06

**Authors:** 


GENTLEMEN,

MR. Jones's case of twins in the last Journal is highly interesting, and I think he displayed much judgment in allowing nature an opportunity of effecting the delivery of the second child.

In the absence of any particular bad symptom, delay cannot be very hazardous, whereas producing delivery "per artem" is always attended with danger both to the mother and her offspring. Our best practical writers on midwifery expressly caution practitioners in that art, not to be hasty and precipitate in doubtful cases. In long protracted labours the greatest difficulty which the accoucheur encounters is the keeping up the confidence of the patient and attendants; when that fails he is sure to be harassed by unceasing requests to do something for her relief, and distressed by doubtful hints as to his ability and knowledge. This often leads him to take a fatal step which may blast his reputation and credit, and furnish him with a never failing source of regret. These observations particularly apply to young professors of the obstetric art, among whom I reckon myself.

In a case which I am about to detail, I condemned myself for rashness, whether justly or not you and your readers may determine. A woman, 22 years of age, under the middle size of females but well proportioned, felt the first pains of child-birth on a Sunday afternoon about the middle of last Summer*. I was desired to attend on the occasion. On my arrival the pains were so slight that I deemed an examination unnessary. During the night the pains continued, but in the most trifling degree, yet they were sufficient to rupture the membrane, and shortly afterwards the pains entirely ceased. Being some miles from home I slept at the house where my patient was confined, and on Monday morning I made my first examination. I found the head presenting in a natural position and very low in the pelvis; I encouraged my patient with the hopes of a speedy delivery on the return of the pains; these I waited for in vain till Tuesday afternoon: I was now prevailed upon by the request of herself and friends, as well as the weak state in which she was, to deliver the child by the forceps. This was the first case in which I ever used that instrument, and I succeeded in delivering the child in a f w minutes with but little pain, considering this was her first pregnan y.

The child was a little under a full grown fœtus, and from her very great size before delivery I immediately judged there were twins; this was really the case. No hæmorrhage succeeded; the placenta was retained by the undelivered child. I continued to encourage my patient with flattering expectations and a little wine, without informing her of the nature of her situation. I waited two hours, no pains returned, nor was there any hæmorrhage, but a great degree of languor. I now introduced my hand into the uterus, ruptured the membranes of the second child, and delivered it by the feet: flooding to an alarming extent instantly succeeded. With as much promptitude as possible I brought away two placentæ, being obliged for this purpose to introduce my hand a second time into the uterus. The gush of blood was now such as I had never before witnessed; the woman exhibited every symptom of mortal syncope, not a trace of pulsation remaining. I was prepared with cloths wetted with brandy and vinegar, and having thrown off the bed cloaths excepting the sheet, I instantly covered the l ins, pubes, and abdomen with them, changing them frequently for others. The flooding soon abated, but this arose from the sunk state of the patient, who had, in a few moments, lost not less than 6 or 7 pounds of blood. For some minutes scarcely a vestige of life was apparent; every attendant had left me excepting one; with her assistance I carefully administered cordials, and my pleasure was great at finding a gradu l return of life. The flooding continued in

* Having lately changed my residence, I am obliged to narrate the case from recollection, my notes being left behind.

a trifling degree for a short time, and then altogether ceased. Good nursing and attention gradually restored the woman.

Was this case to occur to me again I would deliver the first child "per artem," for the great distension of the uterus connected with the weak state of the woman rendered the return of labour pains improbable; but after the delivery of the first child, the uterus being relieved, and no very bad symptom intervening, I might safely, I think, have waited, and allowed nature a reasonable time to have resumed her efforts.

I am, Gentlemen,

Your most obedient servant,

AN ESSEX PRACTITIONER.

*April* 14, 1811.

---

